# Lithium Monitoring Within NHS Forth Valley 2020

**DOI:** 10.1192/bjo.2022.283

**Published:** 2022-06-20

**Authors:** Amy Carbarns

**Affiliations:** Forth Valley Royal Hospital, Larbert, United Kingdom

## Abstract

**Aims:**

The aim of this audit is to improve the monitoring of appropriate parameters in patients within the health board who are prescribed lithium, including lithium level, urea and electrolytes, thyroid function tests, calcium. It will also look at whether the same patients are appropriately referred to renal services.

**Methods:**

A retrospective review of electronic patient records of 100% of the patients in Forth Valley Health Board who have collected a lithium prescription in the 6 months between March and August 2020.

**Results:**

69% of patients had a lithium level checked within the time period set out in the NICE guideline. Only 43% complied with the guideline on renal referral and 63% on calcium. Compliance with assessment of urea and electrolytes was better at 90%, and thyroid function tests at 85%.

**Conclusion:**

Part of the decline in compliance with guidelines is likely in relation to the availability of face-to-face appointments during the pandemic, and reduction in outpatient appointments. As a result of this there is a planned further audit looking at how lithium monitoring is reviewed in outpatient psychiatry. This is intended to increase the involvement of psychiatry and the patient in ensuring appropriate monitoring is completed rather than relying solely on the GP.

